# Heart Rate Variability During Physical Exercise Is Associated With Improved Cognitive Performance in Alzheimer's Dementia Patients—A Longitudinal Feasibility Study

**DOI:** 10.3389/fspor.2021.684089

**Published:** 2021-07-15

**Authors:** Svenja Schwarck, Nancy Busse, Gabriel Ziegler, Wenzel Glanz, Andreas Becke, Emrah Düzel

**Affiliations:** ^1^Institute of Cognitive Neurology and Dementia Research, Otto-von-Guericke-University Magdeburg, Magdeburg, Germany; ^2^German Center of Neurodegenerative Diseases (DZNE), Magdeburg, Germany

**Keywords:** heart rate variability, cognition, cardiovascular training, Alzheimer's disease, neurovisceral integration model, dual task

## Abstract

Heart rate variability (HRV) rapidly gains attention as an important marker of cardiovascular autonomic modulation. Moreover, there is evidence for a link between the autonomic deficit measurable by reduced HRV and the hypoactivity of the cholinergic system, which is prominently affected in Alzheimer's disease (AD). Despite the positive influence of physical exercise on cognition and its promising association with HRV, previous studies did not explore the effect of long-term physical exercise in older adults with AD. Taking advantage of a longitudinal study we analyzed the effect of a 20-week dual task training regime (3 × 15-min per week) on the vagal mediated HRV index RMSSD (root mean square of successive RR interval differences) during physical exercise and the short-term memory performance in a AD cohort (*N* = 14). Each training contained physical exercise on a bicycle ergometer while memorizing 30 successively presented pictures as well as the associated post-exercise picture recognition memory test. Linear-mixed modeling revealed that HRV-RMSSD significantly increased over the intervention time. Moreover, the reaction time in the picture recognition task decreased while the accuracy remained stable. Furthermore, a significantly negative relationship between increased fitness measured by HRV-RMSSD and decreased reaction time was observed. This feasibility study points to the positive effects of a dual task regime on physical and cognitive fitness in a sample with impaired cognitive performance. Beyond this, the results show that the responsiveness of parasympathetic system as measured with HRV can be improved in patients with dementia.

## Introduction

Regular physical exercise across lifespan positively affects cardiovascular health and cognitive function (Cotman and Berchtold, 2002; Gomez-Pinilla and Hillman, 2013). There are promising indications that it operates neuroprotectively by reducing the risk for neurodegenerative diseases (Warburton et al., 2006; Ahlskog et al., 2011; Erickson et al., 2011). The cardiovascular system is regulated by the autonomic nervous system (ANS) (Gordan et al., 2015). Indicator of the cardiac autonomic control is the flexible interaction of the two branches of the ANS's influence on the sinoatrial node, which are the parasympathetic (PNS) and sympathetic (SNS) nervous system (Gordan et al., 2015; Ernst, 2017). The sinoatrial node is characterized by an independent intrinsic heart rate (HR) due to spontaneous depolarization of the membrane (Shaffer et al., 2014; Ernst, 2017). The PNS slows down the spontaneous depolarization and increases the heart rate variability (HRV) due to the release of acetylcholine binding to muscarinic M2 receptors (Shaffer et al., 2014; Ernst, 2017). In contrast, the SNS releases norepinephrine, which binds to beta-1 adrenergic receptors, thereby raising the intrinsic HR and reducing the HRV consequently (Shaffer et al., 2014; Ernst, 2017). HRV is defined as the physiological variation in the time intervals between consecutive R waves (R-R intervals in ms) (Akselrod et al., 1981; Malliani et al., 1991). A common HRV index associated with parasympathetic activity at rest is the RMSSD (root mean square of successive R-R interval differences, in ms), which is a proxy for the vagal mediated variation in HR (Thayer and Lane, 2000; Shaffer et al., 2014).

The present literature showed some evidence that HRV function as a valid indicator of the interaction of PNS and SNS and might serve as a useful marker of cardiovascular autonomic modulation (Shaffer et al., 2014; Ernst, 2017). In line with this, physical exercise directly affects the balance of the two branches, whereby a curvilinear parasympathetic withdrawal was observed in response to increased exercise intensity (Tulppo et al., 1996; Lunt et al., 2011). Consequently, restoring the parasympathetic baseline level after physical exercise takes some time (Buchheit et al., 2007; Stanley et al., 2013). However, the reactivation of PNS can be improved by regular physical exercise (Yamamoto et al., 2001; Seiler et al., 2007; Stanley et al., 2013). As a result, higher physical fitness affects the general autonomic regulation and even causes increased parasympathetic dominance at rest (Carter et al., 2003; Tulppo et al., 2003; Danieli et al., 2014). Especially endurance training appeared to be effective (Sztajzel et al., 2008). In this regard, Sandercock et al. (2005a) showed medium to large effect sizes regarding the overall changes in RR-intervals (*d* = 0.75, *N* = 298) as well as the increased parasympathetic modulation (*d* = 0.48, *N* = 322). However, it should be mentioned that regular physical exercise and associated higher fitness not only impact vagal tone, but also lead to hemodynamic changes, which have a positive impact on the cardiac efficiency (for review see Hawley et al., 2014; Green et al., 2017). Apart from regular physical exercise, there are various important non-modifiable factors influencing HRV, such as age (Thayer et al., 2010). Thayer et al. (2010) showed an overall age-related reduction of cardiac autonomic function. Besides, RMSSD has been expected to decrease about 3.6 ms per decade so that it is significantly reduced in older compared to young and middle-aged adults (Tulppo et al., 1998; Antelmi et al., 2004). Literature showed some evidence, however, that regular physical exercise also significantly increases HRV at rest in healthy older participants (≥60 y) (Levy et al., 1998; Albinet et al., 2010; Soares-Miranda et al., 2014). Therefore, the reduction in RMSSD over age can be countered by regular physical exercise. Not only effects on PNS can be expected after physical exercise, but also effects on the cognitive performance. Studies including healthy young and older adults showed a direct association between reduced HRV at rest and worsened cognition in several domains including memory, attention, and executive functions (Hansen et al., 2003; Kimhy et al., 2013; Gillie et al., 2014; Williams et al., 2016; Colzato and Steenbergen, 2017; Colzato et al., 2018; Ottaviani et al., 2019).

Thus, in addition to the observed exercise-induced fitness benefits, regular physical exercise also has a positive effect on cognition in young and older adults (Hamer and Chida, 2009; Liu-Ambrose et al., 2018). This is why physical exercise is also discussed as a preventive measure for age-related cognitive decline and neurodegeneration (De la Rosa et al., 2020). A prominent neurological disease associated with cognitive decline is dementia (Weller and Budson, 2018). The most common dementia type including at least 60% of all cases is Alzheimer's disease, characterized by a progressive decrease of behavioral and cognitive functions, such as memory, attention, and reasoning (Takizawa et al., 2015; Crous-Bou et al., 2017). Interestingly, a low vagal activity measured by decreased HRV also seems to be a sensitive biomarker of cognitive impairment and cardiovascular as well as neurological diseases (Martin et al., 1987; Cripps et al., 1991; Melo et al., 2005; Vanderlei et al., 2009; Thayer et al., 2010; Ramos Bernardes da Silva Filho et al., 2017; Forte et al., 2019). Accordingly, several dementia subtypes, such as Alzheimer's disease, were found to be associated with reduced HRV indexes reflecting lower parasympathetic activity at rest (Ramos Bernardes da Silva Filho et al., 2017). This autonomic deficit seems to be linked to a hypoactivity of the cholinergic system, which influences both branches of the ANS (Coyle et al., 1983; Perry et al., 1990). Furthermore, the severity of the parasympathetic autonomic dysfunction might be related to the progression of neuropsychological deficits in dementia (Collins et al., 2012). In accordance with the Neurovisceral Integration Model Thayer and Lane (2000), diseased prefrontal-subcortical circuits seem to be linked to reduced cardiac vagal tone (Thayer and Lane, 2000, 2009; Sakaki et al., 2016).

Similar to healthy older adults, positive effects of regular physical exercise alone or embedded in a dual task training on fitness levels and cognitive performance were also found in patients with Alzheimer's disease (Hamer and Chida, 2009; Öhman et al., 2016; Liu-Ambrose et al., 2018). Yet, the majority of these studies used other cardiovascular fitness marker than HRV, such as Vo_2max_, lactate or the resistance (Watt). As HRV is however a promising indicator for fitness as well for cognitive performance (Ramos Bernardes da Silva Filho et al., 2017; Forte et al., 2019), longitudinal physical exercise studies examining its influence on cognition in older adults with cognitive decline are still missing. Therefore, this feasibility study was designed to examine the influence of a longitudinal 24-week bicycle ergometer physical exercise training on the HRV measured during exercise and the visual short-term memory performance in a sample with Alzheimer's dementia. Correspondingly, this study investigated the effect of long-term physical exercise on (i) PNS activity in individuals with Alzheimer's dementia and associated decreased HRV *per se* (ii) visual short-term memory performance, and (iii) and the relationship between vagal activity and visual short-term memory performance. As such, an exercise-induced increase of the parasympathetic index RMSSD and associated positive influence on cognitive performance were hypothesized.

## Materials and Methods

### Participants

Nineteen older adults with diagnosed incipient to moderate dementia in Alzheimer's disease (F00.1) were recruited during September 2017–September 2018 from the memory clinic of the German Center for Neurodegenerative Diseases (DZNE), Magdeburg. Five participants had to be excluded from further analyses (one due to physical problems, four due to technical problems during the data acquisition) resulting in a total sample size of *N* = 14 (age: *M* = 74.07, *SD* = 3.1; Mini Mental Status Examination: *M* = 23.21, *SD* = 3.47; female: *n* = 5). All participants were free of cardiovascular (checked by resting electrocardiogram before the first training session) or pulmonary disorders and symptoms of depression (Geriatric Depression Scale: *M* = 2.0, *SD* = 1.2).

All participants and their relatives as representative signed a written informed consent form for participation. The study was approved by the ethics committee of the Otto-von-Guericke University, Magdeburg, Germany (approval number: 68/17). The study was registered as a clinical trial after the enrolment of participants started since this study was planned as a feasibility study (DRKS registration number: DRKS00019105). The authors confirm that all ongoing and related trials for this intervention are registered. The participants were not compensated monetarily for the costs of participation.

### Experimental Design and Procedure

The study was designed as a 24-week physical and cognitive dual task intervention without a follow-up phase. Three times per week the participants trained for 15 min in their own household. Each dual task training contained a physical exercise training on a bicycle ergometer while simultaneously memorizing pictures presented on an integrated Tablet. After the exercise session, ratings of perceived exertion were collected using a 6–20 Borg Scale (Borg, 1982). Furthermore, a picture recognition memory test assessing visual short-term memory performance followed. The HR and HRV were assessed continuously during the training using a Garmin chest belt. To control circadian rhythm, training sessions were performed on the same days of the week and the same time per participants. Additionally, the MMSE (Mini-Mental State Examination) was assessed in a more flexible time range before (ranging from 12 days to 8 weeks) and immediately after the end of the 24-week intervention.

### Dual Task

The self-developed dual task contained simultaneous physical and cognitive stimulation. Each participant received an individual physical exercise protocol for the regulation of the power output (bike_Power_) calculated as a ratio of resistance and rotations. The required continuous rotation of the physical exercise session was set to 40–80 rotations per minute. The exercise intensity depended on the individual participant's target HR, computed using the Karvonen method (Karvonen et al., 1957). Thus, the participant's target zone was set to 65–75% maximum HR (~90–115 bpm), which was calculated by the age-predicted maximum HR equation 220-age (Tanaka et al., 2001). Using a fixed individual start resistance, the intensity was successively increased each 60s until the target HR was reached. Individual adaptation of the training protocol was accomplished by: (i) examination of the average target HR and (ii) ratings of perceived exertion after each exercise session. Accordingly, the exercise intensity was increased individually over the entire intervention time while the target HR remained approximately the same.

The cognitive stimulation in the dual task was realized by the simultaneous presentation of 30 pictures e.g., landscapes or animals on a tablet (Samsung Galaxy Tab A 2016, 10.1 inch) while exercising. The pictures were presented for 20 s each with a 10 s inter-stimulus interval in which the screen's background turned white. Each training week and the associated three training sessions within this week contained the same 30 pictures, while the pictures differed between the weeks. The average difficulty level of each weekly picture set, determined by the open source LaMem score evaluating image memorability (Khosla et al., 2015), was equal.

Immediately after each dual task training session the picture recognition memory test followed in which 30 test screens were presented one after the other. Each test screen contained an original picture which was visible during the training and a lure picture which was completely new but similar to the memorability score of the original picture. The pictures were presented one above the other, the order (top or bottom) of the original and lure picture was randomized per test screen. For each of the 30 test screens, the participant had to decide one after the other, by touching the picture, which of the two pictures was the original picture. The picture recognition task was designed as a forced-choice format. There was no time limit on the picture response. A training week also contained the same lure pictures, while these differed between the weeks. On average the LaMem memorability score (Khosla et al., 2015) between the week lure sets was equal just as between each lure and original set per week.

### Heart Rate Variability

The HR and associated HRV were assessed during each 15-min physical exercise session in an upright position on a bicycle ergometer. During the whole training sessions participants breathed spontaneously without any control of the frequency or depth of their respiration. The RR intervals for each training session were received continuously from a Garmin chest belt using an ANT+ sensor. HRV data was analyzed according to the recommendation of the Task Force of the European Society of Cardiology and the North American Society of Pacing and Electrophysiology (Task Force of the European Society, 1996). The HRV parameters were analyzed using the R package RHRV (Version 4.2.5) (García Martínez et al., 2017). An automatic filter algorithm was used for the detection and removal of artifacts on the basis of an adaptive threshold for the rejection of RR values that differ from a threshold value based on previous and following beats (Rodríguez-Liñares et al., 2011). Additionally, all RR series were visually examined and adjusted for ectopic heart beats defined as extra beats not originating from the sinoatrial node (Sapoznikov et al., 1992). The artifact threshold was set at 5%. For the calculation of the time domain HRV indexes, a window size of 128 s and a bin width of 7.8125 ms was used resulting in a total number of 7 windows per session.

The subsequently calculated HRV parameter of the time domain was the parasympathetic parameter RMSSD (in ms) as the index for vagal tone (Thayer and Lane, 2000). The RMSSD is the square root of the mean of the squares of differences between adjacent RR intervals. Moreover, the mean of all RR intervals (mean RR, ms) were computed. Both HRV indexes were calculated for each training session.

### Statistical Analysis

The analysis of the physical fitness was conducted using the vagal mediated HRV parameter RMSSD (ms) (Thayer and Lane, 2000). With respect to the post-exercise picture recognition memory, the main cognitive outcomes were the accuracy in percentage correct and the reaction time in seconds. For each participants and each outcome parameter, the average over 1 week containing all three training sessions were calculated. Accordingly, all incomplete training weeks (<3 training sessions) due to due to technical problems regarding the chest belt and/or general physical problems were excluded. The resulting measurement time points at which all participants had complete data were week 1, week 6, week 11, week 16, and week 20. Thus, five measurement time points were included for further analysis resulting in an overall intervention time of 20 weeks, respectively, 5 months.

All statistical analyses were conducted in R version 4.0.2 using RStudio version 1.3.1056 with the packages lme4 (Bates et al., 2015), psych (Revelle, 2020), rstatix (Kassambara, 2020), and influence.ME (Nieuwenhuis et al., 2012). The 2D graphs were created with the R package ggplot2 included in the package tidyverse (Wickham et al., 2019). If required, the assumption of normal distribution was tested using the Shapiro-Wilk Normality test and by visual inspection of histograms of the residuals and Q-Q plots. Data points ± 1.5 multiplied by the interquartile range were defined as outliers and removed from further analysis. The alpha level for all statistical tests was defined as *p* < 0.05.

A paired samples *t*-test was used to analyze the difference of the MMSE before and after the intervention. Due to one missing post MMSE score, one participant was excluded (*n* = 13). Effect size was assessed using Cohen's d. Pearson correlations were calculated for assessing the relationship between the pre MMSE score and the cvRMSSD of week 1 and the post MMSE score and the cvRMSSD of week 20. Two participants were identified as extreme outliers (data points ± 3 multiplied by interquartile range) and excluded. Accordingly, the sample size in these analyses was *n* = 12. Effect size was assessed using the correlation coefficient *r*.

Pearson or Spearman correlations were calculated for assessing the relationship between the parasympathetic HRV parameter RMSSD and the HR at each measurement time point. To remain in the same metric, the meanRR in milliseconds was used instead of the HR in bpm. In the case of a significantly linear relationship, RMSSD was adjusted for the influence of meanRR by calculating the coefficient of variation cvRMSSD, which is RMSSD divided by meanRR multiplied by 100 (van Roon et al., 2016). In addition, Spearman correlations were calculated to examine the relationship between RMSSD and the for meanRR adjusted cvRMSSD. All calculated correlations were 2-sided. The multiple comparisons were Bonferroni adjusted.

Linear mixed effects modeling (LME) was used to analyze the longitudinal data across the five measurement time points. Prior to this, the assumptions for each model were checked. Therefore, the linearity of the relationship between predictor and response and homoscedasticity were examined visually using a residual plot. The normality of the residuals was checked visually *via* a histogram of residuals and as Q-Q plot. Moreover, the data was analyzed for influential data points. As a measure of influence, Cook's distance were calculated using the cut-off value 4 divided by the number of groups (Van der Meer et al., 2010). In the case of influential data points, the LME with and without the influential data points was run and compared regarding the direction and interpretation of the results. Values that significantly change the slope were excluded for further analysis. No influential data points had to be excluded.

Overall, four models were calculated separately. Firstly, for RMSSD as fitness indicator and associated index of vagal tone, secondly for meanRR, thirdly for the cognitive short-term memory performance outcome reaction time in seconds and fourthly for accuracy in percentage correct. To account for individual differences in fitness and cognitive levels, respectively, a random intercept and random slope model was calculated for each of the four models. The included predictor variables were the measurement time point (week 1, week 6, week 11, week 16, and week 20) as the fixed factor and the participants and their associated intercept and slope as the random factor.

Furthermore, to examine the effect of the HRV index RMSSD on both short-term memory outcomes two separate models were computed. Hence, the dependent variable of these models was either reaction time or accuracy and the fixed factor was cvRMSSD. Subjects and their associated intercept were included as the random factor. Effect size estimates (in absolute units of the dependent variable) were assessed using beta coefficients of the LME. Additionally, 95% confidence intervals were calculated.

## Results

### Descriptive Statistics

Table 1 provides the demographic data for the participants. In addition, Table 2 contains the descriptive statistics of the HRV index RMSSD, the adjusted cvRMSSD, the meanRR and the bike_power_ separately for all five measurement time points. Additionally, both parameters of the picture recognition test, accuracy and reaction time, are included in the descriptive statistic table.

**Table 1:** Demographic data and neurological characteristics.

**Demographic data**	
Age (years)	69–80 (*M* = 74.07, *SD* = 3.1)
Sex	Female: *n* = 5, male *n* = 9
**Neurological characteristics**	
ICD-10 diagnosis	Alzheimer's disease (F00.1)
MMSE before	*M* = 23.21, *SD* = 3.47
MMSE after^*^	*M* = 21.92, *SD* = 3.9

*Demographic data and neurological characteristics of the sample (N = 14). MMSE before = Mini Mental Status Examination score before the start of the intervention. MMSE after = Mini Mental Status Examination score after the end of the 24-week intervention*.

* *N = 13 due to one missing value. M, mean, SD, standard deviation*.

**Table 2:** Descriptive statistics of the primary and secondary outcomes.

**Parameter**	**1 M (±SD)**	**2 M (±SD)**	**3 M (±SD)**	**4 M (±SD)**	**5 M (±SD)**
RMSSD	9.89 (4.36)	10.38 (5.70)	11.20 (4.37)	11.33 (6.25)	14.47 (6.37)
cvRMSSD	1.45 (0.59)	1.59 (0.77)	1.72 (0.59)	1.80 (0.96)	2.33 (1.06)
meanRR	671.13 (85.56)	636.56 (68.24)	641.04 (71.28)	638.81 (71.60)	640.53 (75.91)
bike_power_	47.45 (1.67)	61.53 (20.60)	62.67 (20.20)	66.23 (21.66)	67.95 (23.45)
accuracy	83.41 (11.69)	86.93 (10.50)	83.12 (13.38)	83.76 (11.35)	83.37 (14.96)
Reaction time	3.85 (1.14)	2.63 (0.61)	2.78 (0.72)	2.84 (0.69)	2.68 (0.58)

Descriptive statistics of the within-session RMSSD (ms) and adjusted cvRMSSD (coefficient of variation: RMSSD/meanRR *100) as parasympathetic HRV index, the meanRR (mean of all RR intervals, ms) and the bike_power_ (calculated as a ratio of resistance and rotations). Also included are the cognitive outcomes of the picture recognition test accuracy (percentage correct) and reaction time (s). Data are presented for the measurement time points 1 (week 1), 2 (week 6), 3 (week 11), 4 (week 16), and 5 (week 20). Each parameter per measurement time point is the average of the three training session in the corresponding week.

### HRV During Physical Exercise

**Figure 1:**
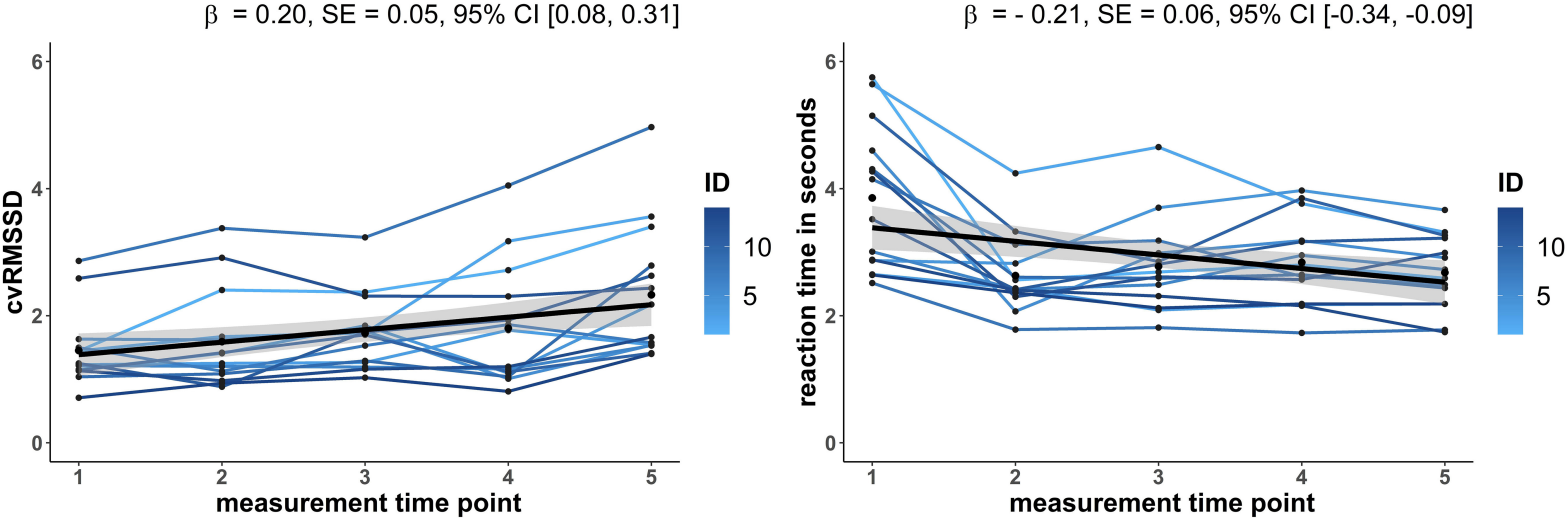
LME results of **(A)** the vagal mediated HRV index cvRMSSD adjusted for meanRR (coefficient of variation: RMSSD/meanRR *100) and **(B)** the reaction time in seconds over the five measurement time points 1 (week 1), 2 (week 6), 3 (week 11), 4 (week 16), and 5 (week 20). The trajectories are shown for each subject (colored solid lines, *N* = 14) and for the estimated group model (black dashed line) with a 95% confidence interval (gray area). Both LME analysis revealed a significant increase, respectively, decrease over the measurement time points. β, beta coefficient of measurement time point effect; *SE*, standard error; 95%-*CI*, 95% confidence interval.

Using Pearson or Spearman correlation, a significantly positive linear relationship between meanRR and RMSSD at week 1 (*r* = 0.60, *p* = 0.029), week 6 (*r* = 0.66, *p* < 0.016), and week 11 (*r* = 0.62, *p* = 0.022) was revealed, while week 16 (*r* = 0.42, *p* = 0.150) and week 20 showed no relationship (*r* = 0.13, *p* = 0.680). After adjusting for the meanRR by calculating the coefficient of variation (cvRMSSD), no significantly positive correlation was observed at week 1 (*r* = 0.31, *p* = 0.30), week 6 (*r* = 0.55, *p* = 0.055), week 11 (*r* = 0.32, *p* = 0.289), week 16 (*r* = 0.14, *p* = 0.66), and week 20 (*r* = −0.19, *p* = 0.451). Furthermore, positive linear relationships were detected between RMSSD and the adjusted cvRMSSD for week 1 (*r* = 0.93, *p* < 0.001), week 6 (*r* = 0.97, *p* < 0.001), week 11 (*r* = 0.92, *p* < 0.001), week 16 (*r* = 0.89, *p* < 0.001), and week 20 (*r* = 0.88, *p* < 0.001). Due to the primarily large relationship between RMSSD and meanRR, the following analysis contains only cvRMSSD adjusted for meanRR. To test the hypothesis that vagal mediated HRV increases over the intervention time, an LME was estimated. A significantly linear increase of the adjusted cvRMSSD [β = 0.20, *SE* = 0.05, 95%-*CI* [0.08, 0.31]] over the measurement time points was observed (Figure 1A). Moreover, a no change of meanRR [β = −5.89, *SE* = 3.60, 95%-*CI* [−13.47, 1.68]] over the intervention time was observed. The corresponding LME results of all analyzed HRV parameter are presented in Table 3.

**Table 3:** LME outcome.

**Index**	**β**	***SE***	***95%-CI***	**Intercept** ***SD***	**Slope** ***SD***
cvRMSSD	0.20	0.05	0.08, 0.31	0.55	0.17
meanRR	−5.89	3.60	−13.47, 1.68	70.96	9.23
Accuracy	−0.32	0.49	−1.31, 0.66	8.83	0.67
Reaction time	−0.21	0.06	−0.34, −0.09	0.83	0.12

Results of the LME analysis of the longitudinal data over the five measurement time points (week 1–week 20). The analysis was calculated for the vagal HRV index cvRMSSD adjusted for meanRR (coefficient of variation: RMSSD/meanRR *100), the meanRR (ms) and the picture recognition test outcome measures accuracy (percentage correct) and reaction time (s). Each LME analysis was calculated separately (N = 14). β, beta coefficient of measurement time point effect; SE, standard error; 95%-CI, 95% confidence interval; Intercept SD, standard deviation of the random intercept; Slope SD, standard deviation of the random slope.

### HRV and MMSE

Furthermore, the paired sample *t*-test revealed no significant change before and after the intervention [*t*_(12)_ = −1.38, *p* = 0.19, 95%-*CI* [−2.58, 0.58], *d* = 0.38, *n* = 13]. In addition, using Pearson correlation, no significant relationship was observed between cvRMSSD and MMSE before [*r* = 0.30, *p* = 0.35, 95%-*CI* [−0.33, 0.74], *n* = 12] and after [*r* = 0.12, *p* = 0.70, 95%-*CI* [−0.48, 0.65], *n* = 12] the intervention.

### Visual Short-Term Memory Performance Over Time

With respect to the picture recognition test outcomes, LME analysis showed a significant linear decrease of reaction time [β = −0.21, *SE* = 0.06, 95%-*CI* [−0.34, −0.09]] over the whole intervention time (Figure 1B). In contrast, accuracy [β = −0.32, *SE* = 0.49, 95%-*CI* [−1.31, 0.66]] showed no linear increase with intervention time. Table 3 also contains the LME analysis results for both picture recognition test outcomes.

### Relationship Between HRV and Visual Short-Term Memory Performance

**Figure 2:**
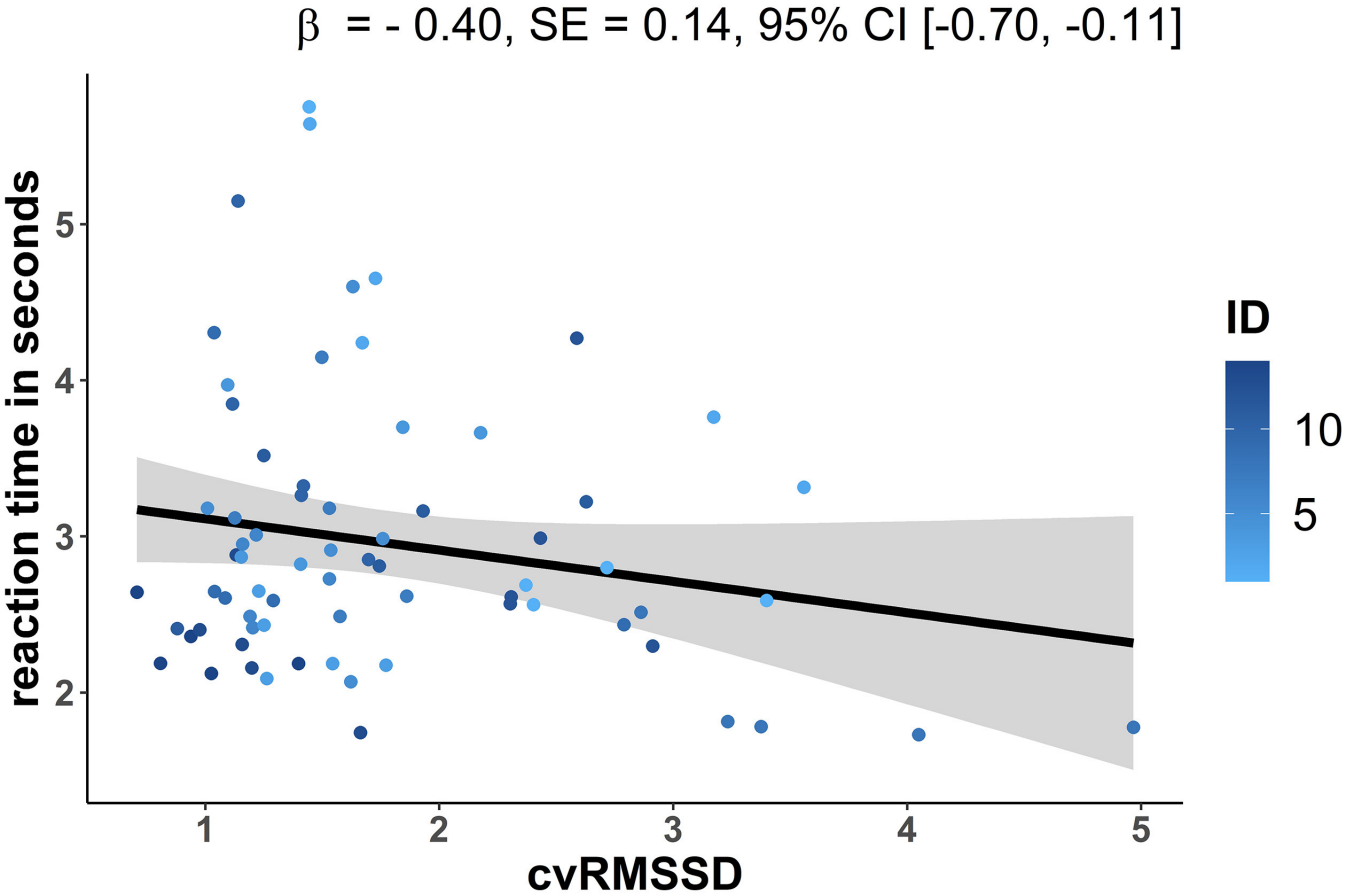
LME results for the model with reaction time (s) as dependent variable (y-axis) and the fixed factor cvRMSSD (coefficient of variation: RMSSD/meanRR *100) on the x-axis. The trajectories are shown for each subject (colored dots, *N* = 14) and for the estimated group model (black dashed line) with a 95% confidence interval (gray area). The LME analysis revealed a significant influence of cvRMSSD on the reaction time. β, beta coefficient of measurement time point effect; *SE*, standard error; 95%-*CI*, 95% confidence interval.

To test the hypothesis of a positive influence of vagal mediated HRV on short-term memory performance, a model including cvRMSSD as fixed factor was calculated separately for reaction time and accuracy (Table 4). A significant linear decrease of reaction time affected by cvRMSSD [β = −0.40, *SE* = 0.14, 95%-*CI* [−0.70, −0.11]] was observed (Figure 2). In contrast, cvRMSSD [β = −0.30, *SE* = 1.32, 95%-*CI* [−2.40, 2.91]] had no significant effect on accuracy.

**Table 4:** LME analysis—short-term memory performance and cvRMSSD.

**Index**	**β**	***SE***	***95%-CI***	**Intercept** ***SD***
Accuracy	−0.30	1.32	−2.40, 2.91	10.80
Reaction time	−0.40	0.14	−0.70, −0.11	0.60

Results of the short-term memory performance and cvRMSSD LME model analysis. The analysis was calculated for reaction time (s) and accuracy (percentage correct) as dependent variable separately (N = 14). The fixed factor was cvRMSSD adjusted for meanRR (coefficient of variation: RMSSD/meanRR *100). β, beta coefficient of measurement time point effect; SE, standard error; 95%-CI, 95% confidence interval. Intercept SD, standard deviation of the random intercept.

## Discussion

The present feasibility study included a 24-week individual aerobic exercise training with simultaneous cognitive stimulation in older adults with Alzheimer's dementia. The study was designed to examine the fitness change of the participants measured with parasympathetic mediated HRV during physical exercise, the change in short-term memory performance assessed regularly post-exercise and the relationship between both outcomes. A significant linear increase over the intervention time and associated fitness effect was observed for the vagal HRV index cvRMSSD (adjusted for the meanRR). Also, improved visual recognition memory performances determined by a significant decrease of reaction time over the whole 20-weeks was found. In contrast, recognition memory accuracy remained stable. Furthermore, a direct positive relationship between fitness and recognition memory performance was observed. Accordingly, increased cvRMSSD affected the decreased reaction time and associated improved cognitive performance over the intervention time. The model included both, measurement time point and cvRMSSD as fixed factors, was significantly more likely compared to the model with measurement time point only. Although, this study was only a feasibility study with a small sample size (*N* = 14) and without a control group, our results suggest some evidence for a positive relationship between exercise-induced increased vagal mediated HRV and improved recognition memory in an older sample with Alzheimer's dementia.

### HRV During Physical Exercise

The present literature showed promising benefits of regular physical exercise on various health aspects such as a neuroprotective effect by reducing the risks for neurodegenerative diseases (Cotman and Berchtold, 2002; Warburton et al., 2006; Ahlskog et al., 2011; Erickson et al., 2011; Gomez-Pinilla and Hillman, 2013). The cardiovascular system is regulated by the flexible interaction of the two branches of the ANS's influence on the sinoatrial node (Gordan et al., 2015; Ernst, 2017). A valid fitness indicator and associated index of cardiovascular autonomic modulation seems to be HRV—a common low-cost diagnostic tool (Gordan et al., 2015; Ernst, 2017). The present study measured the vagal mediated HRV index RMSSD continuously during physical exercise over the entire intervention time. During physical exercise, the parasympathetic HRV proportion withdraws with increasing exercise intensity (Tulppo et al., 1996). Literature showed some evidence, that the decreased RMSSD value is close to minimum during a moderate intensity range (Michael et al., 2017). In our study, each training contained an individual increase of the exercise intensity starting from light to moderate. Accordingly, this pre-defined exercise protocol was in a valid range for the assessment of RMSSD. Moreover, the exercise intensity was increased globally over the entire intervention time using the bike_power_ (calculated as a ratio of resistance and rotations), while the target HR, respectively, the meanRR remained approximately constant. Importantly, this manipulation was also set individually to ensure a valid HRV diagnostic in exactly the measurable range. Correspondingly, despite both manipulations of the bike_power_ and the associated suppressive effect of vagal HRV *per se*, our results showed an increase of parasympathetic activity over the whole intervention time. Accordingly, this exercised-induced shift toward vagal mediated HRV during physical exercise directly shows the associated fitness improvement over time.

Furthermore, the increase of cvRMSSD and the associated fitness effect due to long-term physical exercise in the present study seems to be in line with studies that measured vagal mediated HRV during rest. Regular physical exercise also influences the HRV toward a higher overall variability and parasympathetic dominance at rest while the HR decreases (Sandercock et al., 2005a). Due to the release of acetylcholine binding to muscarinic M2 receptors, the parasympathetic vagus nerve slows down the intrinsic HR (Shaffer et al., 2014). This exercise-induced bradycardia seems to be directly attributable to a shift toward vagal regulation on sinoatrial node measurable *via* HRV indexes at rest (Billman, 2017). In this context, animal studies showed increased atrial acetylcholine concentration and associated increased parasympathetic activity due to a physical exercise training (Herrlich and Raab, 1960; Bolter et al., 1973). Also, a direct stimulation of the vagus nerve on the sinoatrial node seems to impede sympathetic influence (Watanabe et al., 1978; Casado et al., 1994). Nevertheless, cardiovascular fitness also leads to various hemodynamic modifications that are directly accompanied by the increase of vagal mediated HRV at rest (Hawley et al., 2014; Green et al., 2017). For instance, regular physical fitness improves the cardiac output, sheer stress and organic perfusion and lowers the blood pressure at rest (Hawley et al., 2014; Green et al., 2017). Accordingly, exploring the chronological order and mutual influence of these hemodynamic and HRV changes that positively impact cardiac efficiency could be a further research question of interest.

Importantly, in line with the literature during rest (de Geus et al., 2019), the parasympathetic index RMSSD measured during physical exercise also showed a positive linear relationship with the mean of all RR intervals (meanRR). The meanRR was used as a representative of the HR (bpm) to remain in the same metric (ms) as the RMSSD (de Geus et al., 2019). Therefore, RMSSD was adjusted for meanRR using the parsimonious coefficient of variation (cv), which is RMSSD divided by meanRR multiplied by 100 (van Roon et al., 2016). Thus, the metric of both indexes can be maintained and the adjustment also shows no dependency on population-specific slopes or conditions (de Geus et al., 2019). In our results, the adjustment reduced the linear relationship, reflected by decreased correlation coefficients, between RMSSD and meanRR at any measurement time point. Furthermore, the parsimonious coefficient of variation retains the individual rank order on RMSSD (de Geus et al., 2019) which was confirmed in our data due to significant correlations between cvRMSSD and RMSSD at any measurement time point.

However, the mediator of the exercise-induced increase of vagal mediated HRV is still unclear. There is some evidence for suppressed angiotensin II—an inhibitor of cardiac parasympathetic activity (Townend et al., 1995)—in trained compared to less trained subjects presumably reflected by lower levels of plasma renin activity (Fagard et al., 1985; Lijnen et al., 1986). Moreover, there is some evidence that nitric oxide (NO) deduced from neuronal NO synthase modulates the cardiac vagal activity and promotes bradycardia (Travagli and Gillis, 1994; Conlon et al., 1996, 1998). Travagli and Gillis (1994) showed increased cardiac vagal motor neurone activity in the dorsal nucleus of the rat *in vitro* due to the exposure of NO-producing drugs (e.g., L-arginine) while the NO synthase inhibitor L-NAME (N omega-nitro-L-arginine) reduces the excitatory effect. Additionally, stimulation of the vagus nerve in beta-adrenergic blocked ferrets reduces bradycardia after an infusion of L-NAME (Conlon et al., 1996). However, the underlying mechanism is not yet completely understood. Consequently, further research investigating additional biological marker is necessary. An additional HRV measurement during physical exercise could also be considered.

Our results are in line with long-term physical exercise studies showing increased parasympathetic HRV at rest in healthy older adults (≥60 y) (Levy et al., 1998; Albinet et al., 2010; Soares-Miranda et al., 2014). Accordingly, despite the age-related decrease of vagal mediated HRV (Tulppo et al., 1998; Antelmi et al., 2004; Thayer et al., 2010), this decline can be counteracted by regular physical exercise. Beyond that, to the best of our knowledge, this feasibility study was the first to show an exercise-induced increase of cvRMSSD measured during physical exercise in subjects with incipient to mild dementia in Alzheimer's disease. As such, the literature showed a relationship between the development of dementia and overall reduced HRV reflected by decreased parasympathetic indexes and flexibility of the ANS (Thayer et al., 2010; Ramos Bernardes da Silva Filho et al., 2017). In this connection, post mortem studies revealed a link between autonomic dysfunction and the hypoactivity of the cholinergic system, which is well-known to be affected in dementia (Coyle et al., 1983; Perry et al., 1990). Despite the general decline of HRV during age (Tulppo et al., 1998), patients with Alzheimer's disease or mild cognitive impairment showed a significantly stronger decreased PNS activity and increased SNS activity under rest (da Silva et al., 2018). In addition, Giubilei et al. (Giubilei et al., 1998) found a significant correlation between red blood cell acetylcholinesterase and HRV, which may support the link between autonomic dysfunction and cholinergic under activity in the peripheral ANS. Moreover, there is also evidence that vagal mediated HRV and the associated improvement due to regular physical exercise is related to cognition, a domain that is already impaired in the early stages of Alzheimer's dementia (Ramos Bernardes da Silva Filho et al., 2017; Forte et al., 2019).

### Influence of HRV on Cognition

The extent of autonomic dysfunction has been related to the severity of the neuropsychological deficits (Kim et al., 2006; Collins et al., 2012). There is an ongoing discussion regarding a potential relationship between HRV and cognitive performance in different domains (for review see Forte et al., 2019). The PNS contributes to the flexible adaptation and response of rapid changes in environmental demands and reduced cardiac vagal tone seems to directly worsen this ability (Porges, 1995; Thayer and Lane, 2000, 2009). Accordingly, autonomic dysfunction seems to be related to worsened cognitive performance (Thayer and Lane, 2009; Thayer et al., 2010). Vagal mediated HRV, for instance RMSSD, may be linked to different neural structures associated with cognition (Thayer et al., 2010). According to the Neurovisceral Integration Model (Thayer and Lane, 2009; Thayer et al., 2012) the cortical activity directly modulates the autonomic cardiovascular function (Thayer and Lane, 2000, 2009; Thayer et al., 2012). In general, the ANS is under tonic inhibition by cortical circuits between the prefrontal cortex (PFC), cingulate cortex, insula cortices, and the amygdala (Thayer and Sternberg, 2006; Thayer et al., 2012). Those brain areas are critical for cognitive functioning (Critchley et al., 2003) and partly affected by atrophy Alzheimer's disease, also already in early stages (Baron et al., 2001; Bottino et al., 2002; Scahill et al., 2002; Basso et al., 2006; Tan et al., 2013; Ramos Bernardes da Silva Filho et al., 2017). Correspondingly, decreased activation of the interconnected network of PFC, cingulate cortex and insular cortices would lead to a disinhibition of the amygdala and therefore to an activation of sympathoexcitatory circuits in the medulla and associated decrease in HRV (Thayer et al., 2012). Accordingly, hypoactive prefrontal-subcortical circuits and related impaired cognitive performance seem to be directly measurable *via* reduced vagal mediated HRV indexes at rest (Thayer and Sternberg, 2006; Park and Thayer, 2014). In line with this, resting-state fMRI studies with healthy young and older adults confirmed that higher resting vagal mediated HRV is strongly linked to higher functional connectivity of these prefrontal-subcortical circuits (Chang et al., 2013; Jennings et al., 2016; Sakaki et al., 2016).

Our results provide some support for the Neurovisceral Integration Model (Thayer and Lane, 2009; Thayer et al., 2012). Our feasibility study showed a relationship between exercise-induced increased vagal mediated HRV and improved recognition memory performance reflected by faster reaction times. We found that each unit of increased physical fitness reflected by cvRMSSD affects the reaction time by lowering it about 0.4 s. In contrast, no notable general change and no association with HRV was observed for the accuracy, resulting in stable performance over the whole intervention time. The approximately consistent accuracy over all measurement time points could be directly attributable to the general difficulty level of the picture recognition task assessing visual short-term memory performance. During the retrieval the subjects had to decide 30 times between an original and a lure picture, which were presented at the same time. Accordingly, with respect to the accuracy, the task might have been too easy, as the accuracy was already about 83% at the beginning of the intervention. In addition, the MMSE, included as an overall screening measure of cognitive impairment, also remained stable over the intervention time. Moreover, no significant correlation was observed between neither the pre MMSE score and cvRMSSD at week 1 nor between the post MMSE score and cvRMSSD at week 20. Accordingly, in contrast to the literature (Wang et al., 1994; Kim et al., 2006; Collins et al., 2012), our results cannot support a relationship between the severity of Alzheimer's disease using MMSE and parasympathetic HRV. However, our study differ in some respects, such as the length and position of the HRV measurement, which directly affects the HRV indexes (Shaffer et al., 2014). Furthermore, each analysis contained a small sample size (*n* = 13) and between subjects a comparatively long and partly different period of time between the MMSE and HRV assessment pre (12 days−8 weeks) and post (4 weeks), which both could bias the results. Nevertheless, regarding reaction time, our study supports the discussed relationship between parasympathetic HRV and cognition. It should be noted that we can not exclude the possibility that the relationship may be due, or at least partly due, to common state variability (Geiser et al., 2015). Spontaneous time point to time point physiological fluctuations common with the reaction time could be caused by several influences that are hard to control, such the daily mood (Eid et al., 1999; Geiser et al., 2015). More specifically with respect to HRV, influencing factors are for instance physiological (e.g., respiration), neuropsychological (e.g., emotion, stress), or lifestyle (e.g., medication, smoking) aspects (Fatisson et al., 2016). Accordingly, HRV seems to be a relatively time instable measure (Sandercock et al., 2005b; Shaffer et al., 2014; Uhlig et al., 2020), even when the measurement is repeated immediately (Cipryan and Litschmannova, 2013). Nevertheless, with increasing time points the trait variance seems to increase from 49% after one measurement up to 75% after three (Bertsch et al., 2012). Correspondingly, each time point in this study consisting of three training sessions could lead to a more stable HRV index. In addition, our results are in line with various non-exercise studies with healthy subjects showing a positive parasympathetic HRV and cognition relationship (Hansen et al., 2003; Frewen et al., 2013; Kimhy et al., 2013; Gillie et al., 2014; Williams et al., 2016; Colzato and Steenbergen, 2017; Colzato et al., 2018; Ottaviani et al., 2019) in accordance with the Neurovisceral Integration Model (Thayer and Lane, 2009; Thayer et al., 2012). A closer look at these studies reveals, however, that the most frequently investigated cognitive domain are executive functions, which show a primary positive relationship with HRV (Forte et al., 2019). Hence, our results are in line since the successfully execution of a dual task requires certain aspects of executive function (Banich, 2009; Logue and Gould, 2014). Beyond that, studies examining other cognitive domains like memory are underrepresented and the current literature revealed only minor evidence regarding a positive association between HRV and memory (Shah et al., 2011; Frewen et al., 2013; Zeki Al Hazzouri et al., 2014). Furthermore, the results of the present study are consistent with Hansen et al. (2004) showing improved cognitive performance due to a moderate aerobic exercise training and associated higher resting HRV compared to detrained healthy young subjects. However, it should be noted that the study of Hansen et al. (2004) differs from our study in certain aspects like the study sample (young healthy vs. elderly diseased) and the HRV measurement did not take place during physical exercise.

Furthermore, in addition to the observed positive cardiovascular and cognitive effects, the designed intervention also demonstrated a practical applicability and feasibility. Accordingly, the intervention was simple to handle for older adults with Alzheimer's dementia in their own household and relatively inexpensive in comparison to other treatments. Moreover, the intervention also seems feasible for clinical use. Using HRV as a diagnostic tool in combination with the participant's perceived exertion, flexible adaptations of the exercise protocol can be made in order to improve physical and related cognitive fitness individually and also to avoid exercise-induced overload or underload. However, this study sample was too small to provide practical guidance on training management and age- and gender-specific norms. Nevertheless, we recommend an individual adaptation of the fitness protocol, using a longitudinal physical exercise regime to detect cardiovascular and cognitive changes. According to our results, an increase in vagal mediated HRV during physical exercise and improvements in recognition memory would be expected in a comparable sample of older adults with Alzheimer's dementia and a 24-week dual task. However, considering the rapid decline in vagal mediated HRV and cognitive performance in dementia (Collins et al., 2012; Ramos Bernardes da Silva Filho et al., 2017), maintaining both parameters can be considered as a success of the intervention.

To summarize, using parasympathetic HRV as a fitness index, improvement in physical fitness was shown across a 24 weeks intervention in an older sample with Alzheimer's dementia. Our results are in line with previous studies reporting an exercise-induced fitness effect in older adults with Alzheimer's disease (Hamer and Chida, 2009; Liu-Ambrose et al., 2018). We found that cognitive improvement, decreased reaction times in recognition memory, was significantly related to the vagal mediated HRV, which supports the Neurovisceral Integration Model (Thayer and Lane, 2009; Thayer et al., 2012). The results of this feasibility study are remarkable considering (i) the reduced HRV in patients with Alzheimer's dementia (da Silva et al., 2018) and (ii) the observation of an improvement under dual-task demands. Our results show that HRV can be improved in Alzheimer's dementia, suggesting that physical exercise can lead to a recovery of the responsiveness of the parasympathetic system. Whether this responsiveness is directly related to cognitive improvement remains to the established but could be feasible given that both are affected by cholinergic neurotransmission.

## Limitations

This 24-week longitudinal dual task feasibility study has several limitations. First, the homogenous sample size was small (*N* = 14) and biased regarding age and severity of Alzheimer's disease. In addition, our study design did not include a control group so that we have reduced validity due to the lack of comparability with individuals receiving only cognitive or only physical exercise training. Using a longitudinal study design with multiple repeated measurements, however, the associated power strongly increases while the impact of measurement errors decreases compared to cross-sectional studies. Still future randomized controlled studies are strongly encouraged to include an a priori calculation of an appropriate sample size to verify the results of this feasibility study. Moreover, the study design is limited with respect to the measurement of HRV only during physical exercise. Moreover, the persistence of the fitness effect beyond the end of the intervention remains elusive. Future studies should include at least a pre-post cognitive assessment including a variety of cognitive domains and a resting state HRV assessment to verify the results measured during the dual task regime. In addition, our within-subject comparisons might be biased by confounding effects happening alongside the intervention procedure (e.g., increased attention, social interactions). Furthermore, since the intervention was designed as a dual task and therefore included simultaneous physical exercise and cognitive stimulation, the isolated exercise and learning effect cannot be separated from each other. Moreover, due to the small sample size, we did not include participant's Alzheimer's disease related drugs in our analysis that can impact HRV (Eid et al., 1999). Correspondingly, further studies could include drugs as a covariate. Also, we did not control the individual fitness level at the start of the intervention. Nevertheless, using LME differences in HRV between subjects were accounted due to the calculation and comparison of individual random intercepts. Lastly, we did not control respiration, participants were instructed to breathe spontaneously. However, literature showed that a longitudinal moderate intensity training did not significantly affect respiratory rate (Iwasaki et al., 2003). Nevertheless, there is a probability of an exercised-induced shift regarding the rate of respiration and associated effect on HRV indexes, which should be controlled for in future studies.

Although this was a feasibility study, it is to stress that our results show first evidence for a relationship between increased exercise-induced parasympathetic mediated HRV and improved cognitive performance in an elderly sample with Alzheimer's disease.

## Conclusion

The present study might support the Neurovisceral Integration Model (Thayer and Lane, 2009; Thayer et al., 2012) hypothesizing a direct modulation of cortical activity on the autonomic cardiovascular function. Our results showed an exercise-induced increase of vagal mediated HRV and an association with improved cognitive performance over a 20-week long-term physical and cognitive dual task regime in a sample with Alzheimer's disease. The reaction time significantly decreased over the whole intervention time, while the accuracy in the picture recognition task assessing visual short-term memory performance remained stable. Moreover, a direct relationship between the increased physical fitness measured by cvRMSSD and the improved cognitive performance indicated by decreased reaction time was observed. Thus, our findings may support the discussed relationship between vagal mediated HRV and cognitive performance, even in an elderly sample with Alzheimer's disease. Importantly, this feasibility study measured HRV directly during physical exercise and therefore contributes to another research subfield in the discussed relationship between HRV and cognition compared to the primary represented measurement during rest (Thayer et al., 2012). Future randomized controlled studies including an appropriate study size are needed to verify the results of the feasibility study. Therefore, a more comprehensive pre-post cognitive assessment, in addition to the MMSE, including a variety of cognitive domains and a resting state HRV assessment should be taken into account. In addition, factors that influence the HRV, such as mood or the daily form, should be recorded before each training session. Moreover, the inclusion of several degrees of severity of Alzheimer's disease should be analyzed with regard to fitness differences and the progression of clinical symptoms.

## Data Availability Statement

The raw data supporting the conclusions of this article will be made available by the authors, without undue reservation.

## Ethics Statement

The studies involving human participants were reviewed and approved by ethics committee of the Otto-von-Guericke University, Magdeburg, Germany. The patients/participants provided their written informed consent to participate in this study.

## Author Contributions

AB and SS contributed to the conceptualization and the data curation. SS, GZ, and AB performed the formal analysis. ED contributed to the funding acquisition, resources, and the supervision. The investigation was conducted by NB, WG, AB, and SS. ED, AB, GZ, and SS contributed to the methodology and AB and NB to the project administration. The software and validation was conducted by AB. SS contributed to the visualization and the writing of the original draft preparation. SS, NB, GZ, WG, AB, and ED contributed to the writing of the review and editing. All authors contributed to the article and approved the submitted version.

## Conflict of Interest

The authors declare that the research was conducted in the absence of any commercial or financial relationships that could be construed as a potential conflict of interest.
